# Restricting extracellular Ca^2+^ on gefitinib-resistant non-small cell lung cancer cells reverses altered epidermal growth factor-mediated Ca^2+^ response, which consequently enhances gefitinib sensitivity

**DOI:** 10.1371/journal.pone.0238155

**Published:** 2020-08-25

**Authors:** Mi Seong Kim, So Hui Kim, Sei Hoon Yang, Min Seuk Kim

**Affiliations:** 1 Department of Oral Physiology, Institute of Biomaterial-Implant, School of Dentistry, Wonkwang University, Iksan, Republic of Korea; 2 Wonkwang Dental Research Institute, School of Dentistry, Wonkwang University, Iksan, Republic of Korea; 3 Department of Carbon Convergence Engineering, College of Engineering, Wonkwang University, Iksan, Republic of Korea; 4 Department of Internal Medicine, School of Medicine, Wonkwang University, Iksan, Republic of Korea; Seoul National University College of Pharmacy, REPUBLIC OF KOREA

## Abstract

Non-small cell lung cancer (NSCLC), one of the leading causes of cancer-related death, has a low 5-year survival rate owing to the inevitable acquired resistance toward antitumor drugs, platinum-based chemotherapy, and targeted therapy. Epidermal growth factor (EGF)-EGF receptor (EGFR) signaling activates downstream events leading to phospholipase C/inositol trisphosphate (IP_3_)/Ca^2+^ release from IP_3_-sensitive Ca^2+^ stores to modulate cell proliferation, motility, and invasion. However, the role of EGFR-mediated Ca^2+^ signaling in acquired drug resistance is not fully understood. Here, we analyzed alterations of intracellular Ca^2+^ ([Ca^2+^]i) responses between gefitinib-sensitive NSCLC PC-9 cells and gefitinib-resistant NSCLC PC-9/GR cells, and we found that acute EGF treatment elicited intracellular Ca^2+^ ([Ca^2+^]i) oscillations in PC-9 cells but not in PC-9/GR cells. PC-9/GR cells presented a more sustained basal [Ca^2+^]i level, lower endoplasmic reticulum Ca^2+^ level, and higher spontaneous extracellular Ca^2+^ ([Ca^2+^]e) influx than PC-9 cells. Notably, restricting [Ca^2+^]e in both cell types induced identical [Ca^2+^]i oscillations, dependent on phospholipase C and EGFR activation. Consequently, restricting [Ca^2+^]e in PC-9/GR cells upregulated gefitinib-mediated poly (ADP-ribose) polymerase cleavage, an increase in Bax/Bcl-2 ratio, cytotoxicity, and apoptosis. In addition, nuclear factor of activated T cell (NFAT1) induction in response to EGF was inhibited by gefitinib in PC-9 cells, whereas EGF-mediated NFAT1 induction in PC-9/GR cells was sustained regardless of gefitinib treatment. Restricting [Ca^2+^]e in PC-9/GR cells significantly reduced EGF-mediated NFAT1 induction. These findings indicate that spontaneous [Ca^2+^]e influx in NSCLC cells plays a pivotal role in developing acquired drug resistance and suggest that restricting [Ca^2+^]e may be a potential strategy for modulating drug-sensitivity.

## Introduction

The incidence of non-small cell lung cancer (NSCLC) is steadily increasing and accounts for 85% of lung cancer subtypes. Owing to its recurrence, NSCLC has a low 5-year survival rate of <15% [1]. Since the development of first-generation epidermal growth factor receptor (EGFR)-tyrosine kinase inhibitors (TKIs), such as gefitinib, erlotinib, and ecotinib, diverse targeted therapies operating at the molecular and genetic levels have emerged rapidly. Despite these developments, intrinsic or acquired drug resistance to chemotherapeutic agents allows cancer cells to bypass cell death. Overexpression and over-activity of EGFR are observed in >60% of NSCLC cells [2]. Moreover, prolonged treatment with EGFR-TKIs frequently causes EGFR mutations and interferes with its underlying signaling pathways; thus, prolonged EGFR-TKI use limits its clinical efficacy [3].

In the 2019-guideline v3, the National Comprehensive Cancer Network indicates that genes, including *EGFR*, *ALK*, *BRAF*, *KRAS*, *HER2*, *ROS1*, *RET*, and *MET*, are therapeutic targets for treating NSCLC. TKIs targeting these fundamentally inhibit signaling cascades related to cell proliferation, by which cancer cells frequently show acquired drug resistance owing to gene mutations, including rearrangement, amplification, and point mutation [4, 5]. According to the Kyoto Encyclopedia of Genes and Genomes database, EGFR and its underlying mechanisms appear dependent on the intracellular Ca^2+^ ([Ca^2+^]i) signaling pathway in NSCLC, in which EGFR activation sequentially elicits phospholipase Cγ (PLCγ) phosphorylation, inositol trisphosphate (IP_3_) production, Ca^2+^ release from the endoplasmic reticulum (ER), and protein kinase C activation. Acute human glioma cell stimulation with EGF evokes intracellular Ca^2+^ responses as oscillations, which are blocked by EGFR inhibitors [6]. Thus, the correlation between intracellular Ca^2+^ signaling and acquired drug resistance appears to be interactive; however, the role of Ca^2+^ signaling in drug resistance is not fully understood.

Free intracellular Ca^2+^ acts as a second messenger to regulate the proliferation, migration, and apoptosis of cancer cells [7]. Ca^2+^ depletion in the ER mediates Stromal interaction molecule 1 (Stim1)-dependent Ca^2+^ influx through store-operated Ca^2+^ channels (SOCCs), such as ORAI calcium release-activated calcium modulator 1 (Orai1) and (transient receptor potential channels) TRPCs [8]. Altered Ca^2+^ signaling in cancer development and progression has been frequently observed. Increased Ca^2+^ influx through TRPC5 upregulates autophagic flux to prevent cancer-cell death and promotes drug resistance [9]. Anticancer drugs, including cisplatin, 5-fluorouracil, and gemcitabine in osteosarcomas and pancreatic adenocarcinomas, appear to enhance Orai1 and Stim1 expression, which prevents drug-mediated cell death [10, 11]. Notably, treatment using EGFR-targeting afatinib causes Ca^2+^ signaling-related gene expression in PC-9 cells. Reduced extracellular Ca^2+^ ([Ca^2+^]e) levels increase the sensitivity of PC-9 cells to afatinib [12]. These reports strongly suggest that Ca^2+^ signaling in cancer cells is significantly associated with the development of acquired drug resistance. However, the role of EGFR-mediated Ca^2+^ signaling in acquired drug resistance is not fully understood.

In this study, we demonstrated that the EGF-mediated Ca^2+^ response in NSCLC cells was altered depending on gefitinib resistance and [Ca^2+^]e restriction on gefitinib-sensitive and gefitinib-resistant cells elicited identical [Ca^2+^]i oscillations, which were associated with the modulation of gefitinib efficacy.

## Materials and methods

### Cell culture and reagents

The lung adenocarcinoma cell line, PC-9, and its gefitinib-resistant sub-cell line, PC-9/GR possessing the T790M mutation, were gifted by Dr. Jin Kyung, Rho [13]. Cells were cultured in RPMI-1640 (Hyclone, Logan, UT, USA) supplemented with 10% fetal bovine serum (FBS), 100 U/mL penicillin, and 100 μg/mL streptomycin (Gibco) at 37°C in 5% CO_2_. To determine the effects of [Ca^2+^]e restriction, the culture medium was exchanged with RPMI-1640 without CaCl_2_ (MyBioSource, CA, USA) and 1 mM CaCl_2_ was added. Cyclopiazonic acid was purchased from Enzo Life Sciences (Farmingdale, NY, USA). The mouse monoclonal antibodies against nuclear factor of activated T cell (NFAT1; cat # sc-7296), Orai1 (cat # sc-377281), Bcl-2 (cat # sc-509), and Bax (cat # sc-20067) were obtained from Santa Cruz Biotechnology (Dallas, TX, USA). U-compounds (U73122 and U73343) were obtained from Sigma Aldrich (St. Louis, MO, USA). Human EGF, gefitinib, and rabbit polyclonal antibodies against poly (ADP-ribose) polymerase-1 (PARP; cat #9542), stromal interaction molecule 1 (STIM1; cat #5668), sarco/endoplasmic reticulum Ca^2+^-ATPase 2 (SERCA2; cat #4388), and β-actin (cat #4967) were obtained from Cell Signaling Technology, Inc. (Danvers, MA, USA).

### Measurement of [Ca^2+^]i

The [Ca^2+^]i was measured using a Ca^2+^-sensitive fluorescence dye, Fura-2/AM (Sigma Aldrich) as described previously [14]. Briefly, cells were plated on coverslips at 80% confluence and loaded with Fura-2/AM (5 μM) for 1 h at 37°C in a 5% CO_2_ incubator. Cells were perfused with HEPES buffer containing 140 mmol/L NaCl, 5 mmol/L KCl, 1 mmol/L MgCl_2_, 1 mmol/L CaCl_2_, 10 mmol/L HEPES, and 10 mmol/L glucose. The pH and osmolarity were adjusted to 7.4 and 310 mOsm. Ca^2+^-free HEPES buffer was replaced with 1 mmol/L EGTA. Following brief washing with HEPES buffer, trapped intracellular Fura-2 was excited at 340 and 380 nm. Emitted fluorescence at 510 nm was captured using a charge-coupled device camera. Images were analyzed using MetaFluor software (Molecular Devices, San Jose, CA, USA) and presented as F340/F380 ratio.

### Cell viability assay

Cell viability was determined using EZ-CYTOX (Daeil Lab Service Co. Ltd., Seoul, South Korea), following the manufacturer’s procedure. In brief, cells were treated with EZ-Cytox (10 μL) in each well and incubated for 30 min. Cell viability was measured at OD 450 nm using the iMAX Microplate Reader (Bio-Rad, Hercules, CA, USA).

### Cytotoxicity assay

Cytotoxicity was determined by measuring extracellular glucose-6-phosphate dehydrogenase (G6PD) levels using a Vybrant cytotoxicity assay kit (Invitrogen, Carlsbad, CA, USA) following the manufacturer’s protocols. Briefly, cells were seeded onto 96-well plates and incubated under indicated conditions. Cell culture medium without cells was collected and G6PD activity was determined at excitation and emission wavelengths of 544 and 590 nm, respectively. The result is expressed as the percentage of total G6PD detected in cell lysates from parallel wells.

### Flow cytometry

The early apoptosis rate was determined using the FITC Annexin V Apoptosis Detection Kit with PI (BioLegend, San Diego, CA, USA) following the manufacturer’s protocol. In brief, after a 24-h incubation, cells were washed with Cell Staining Buffer and resuspended with the Annexin V-FITC and PI mixture for 15 min at room temperature in the dark. After adding Annexin V Binding buffer, a minimum of 10,000 cells were analyzed using FACScan analyzer (Becton Dickinson, Franklin Lakes, NJ, USA).

### Western blot

Protein levels were determined using western blotting [15]. In brief, cells were lysed with RIPA buffer (Invitrogen) containing proteases and a phosphatase inhibitor cocktail (Invitrogen). Total lysates were loaded on sodium dodecyl sulfate-polyacrylamide gel electrophoresis gel, separated using gel electrophoresis, transferred onto polyvinylidene fluoride membranes (0.2 μm pore size), and incubated with primary antibodies PARP (1:1000), Bax (1:1000), Bcl-2 (1:1000), and NFAT1 (1:1000); horseradish peroxidase-conjugated IgG was used as the secondary antibody. Immunoreactive proteins were detected using AzureSpot 2.0 (Azurebiosystems, CA, USA).

### Statistical analysis

Statistical analysis was conducted using Origin 2020 software (OriginLab Corporation, MA, USA). Data are presented as the mean ± standard deviation of observations obtained from more than three independent experiments. Statistical differences were analyzed using one-way ANOVA followed by Tukey’s post hoc test and T-test. Values of *p* < 0.05 were considered statistically significant.

## Results

### Basal level of [Ca^2+^]i in PC-9/GR cells is sustained by spontaneous extracellular Ca^2+^ influx, resulting in abolishment of EGF-mediated [Ca^2+^]i oscillations

**Fig 1:**
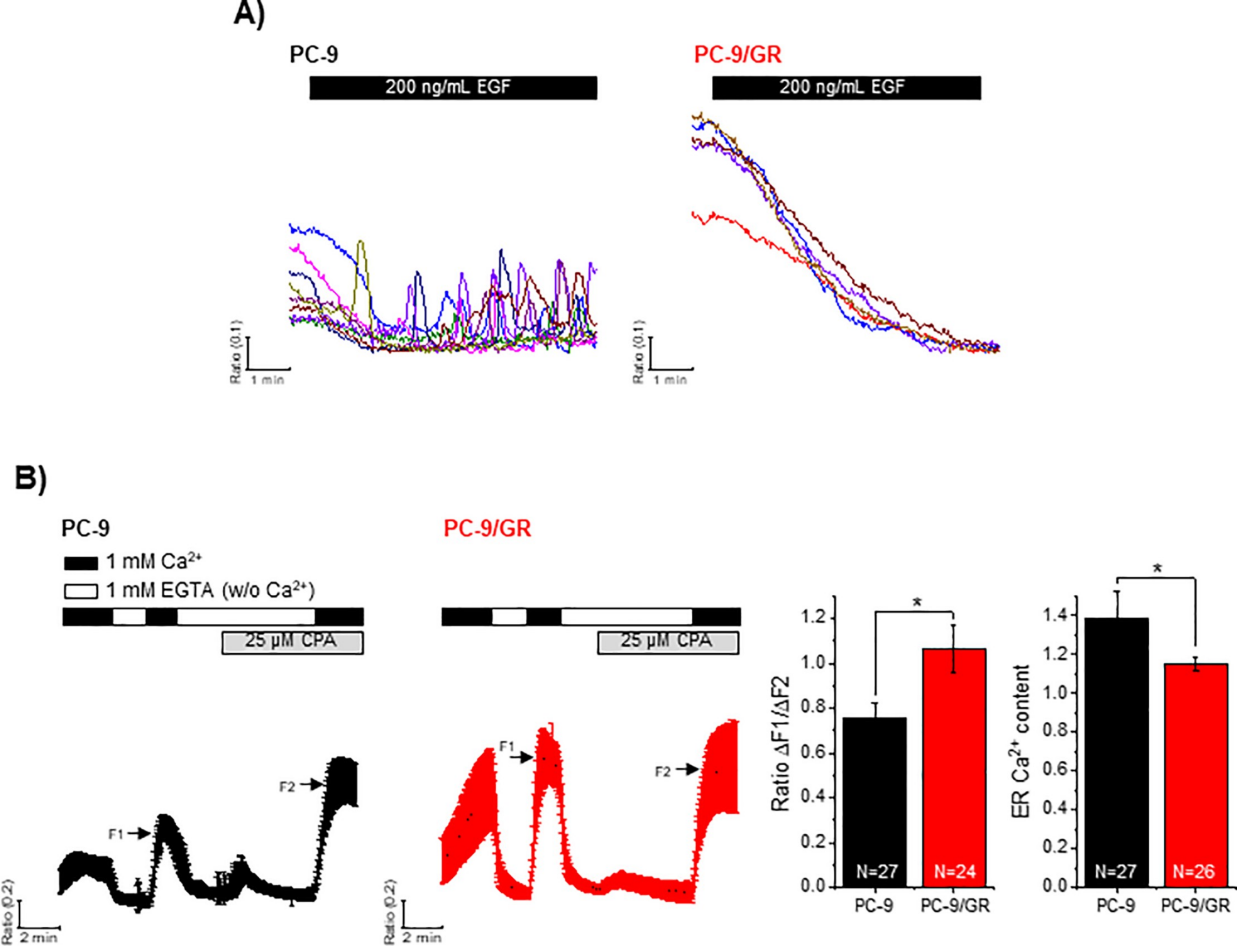
Differential [Ca^2+^]i responses between PC-9 and PC-9/GR cells. Intracellular free Ca^2+^ in live single cell was determined using Fura-2/AM fluorescent dye. (A) EGF-mediated [Ca^2+^]i responses were measured in PC-9 and PC-9/GR cells. Each trace represents [Ca^2+^]i mobilization in a single cell. (B) ER Ca^2+^ content, spontaneous [Ca^2+^]e influx (ΔF1), and [Ca^2+^]i entry via SOCC (ΔF2) were determined is response to CPA (indicated as F1) and rescue of [Ca^2+^]e (indicated as F2) in PC-9 and PC-9/GR cells. The columns show the mean ± S.D. *P < 0.05.

To determine altered EGF-mediated [Ca^2+^]i responses between PC-9 and PC-9/GR cells, we performed a ratiometric assay using Fura-2/AM. Acute treatment with 200 ng/mL of EGF induced [Ca^2+^]i oscillations in PC-9 cells but not in PC-9/GR cells (Fig 1A). PC-9/GR cells showed a more highly sustained basal level of [Ca^2+^]i than PC-9 cells; thus, we examined the spontaneous Ca^2+^ influx in both cell types. To evaluate the spontaneous [Ca^2+^]e influx and determine ER Ca^2+^ content, cells were exposed to Ca^2+^-free HEPES buffer and HEPES buffer (1 mM Ca^2+^). Cells were treated with cyclopiazonic acid to deplete ER Ca^2+^. The spontaneous Ca^2+^ influx (indicated as F1) was greater in PC-9/GR cells than in PC-9 cells, and ER Ca^2+^ content in PC-9/GR cells was 15% lower than that in PC-9 cells (Fig 1B). We additionally characterized the expression of 3 different genes, Orai1, STIM1, and SERCA2, which are essential for mediating store-operated Ca^2+^ entry (SOCE). Expression of dimeric Orai1 and STIM1 showed no significant difference between PC-9 and PC-9/GR cells, whereas SERCA2 in PC-9/GR cells was significantly reduced by about 35% compared to PC-9 (S1 Fig). These results suggest that drug-resistant tumor cells somehow develop altered intracellular Ca^2+^ signaling, which may act as a bypass to activate downstream signals for cell survival.

### Altered EGF-mediated [Ca^2+^]i response in PC-9/GR cells is reversed in an identical manner to that of PC-9 cells by restricting [Ca^2+^]e

**Fig 2:**
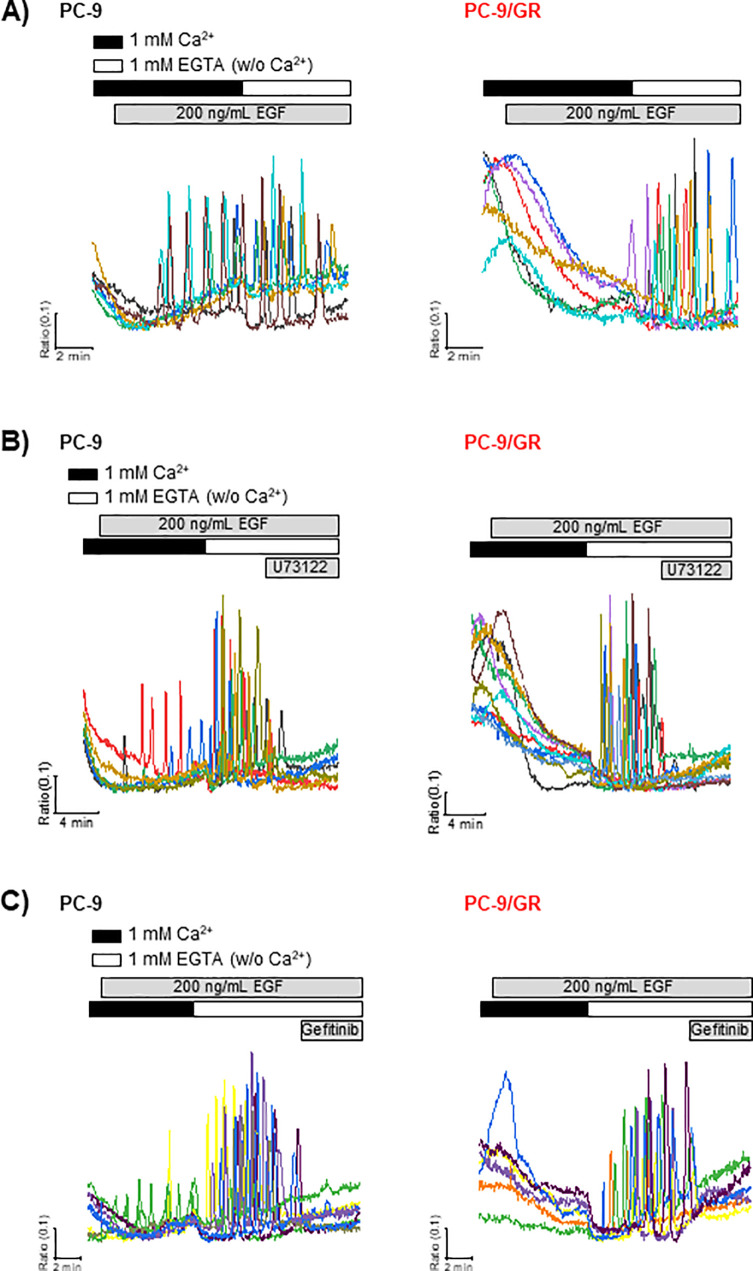
Characterizations of low [Ca^2+^]e-mediated [Ca^2+^]i oscillations. PC-9 and PC-9/GR cells loaded with Fura-2/AM were used to determine [Ca^2+^]i in response to low [Ca^2+^]e, U73122, and gefitinib. (A) Induction of [Ca^2+^]i oscillations in response to low [Ca^2+^]e in EGF-stimulated cells. (B) 2 μM of U73122, an inhibitor of PLC, and (C) 0.1 μM of gefitinib diluted in Ca^2+^-free HEPES buffer were acutely treated on EGF-stimulated cells. Trace represents each [Ca^2+^]i mobilization in a single cell during the indicated time.

Consequently, we examined the effects of [Ca^2+^]e restriction on EGF-stimulated PC-9 and PC-9/GR cells. EGF-mediated [Ca^2+^]i oscillations in PC-9 cells lasted even in the absence of extracellular Ca^2+^ (Fig 2A). PC-9/GR cells somehow exerted [Ca^2+^]i oscillations upon exposure to Ca^2+^-free HEPES buffer, which was in a manner identical to that in PC-9 cells. Based on reports that EGF-EGFR signaling activates PLC and [Ca^2+^]i mobilization [16], we examined whether the [Ca^2+^]i oscillations evoked by [Ca^2+^]e restriction were dependent on PLC and EGFR activation. Low [Ca^2+^]e-induced [Ca^2+^]i oscillations were abolished by U73122 (2 μM), an inhibitor of PLC, and gefitinib (0.1 μM) in PC-9 and PC-9/GR cells (Fig 2B and 2C). These results suggest that restricting [Ca^2+^]e may revert the cytotoxic effects of gefitinib on PC-9/GR cells.

### [Ca^2+^]e restriction and PLC inhibition synergistically exacerbate gefitinib-induced cytotoxicity and cell viability in PC-9/GR cells

**Fig 3:**
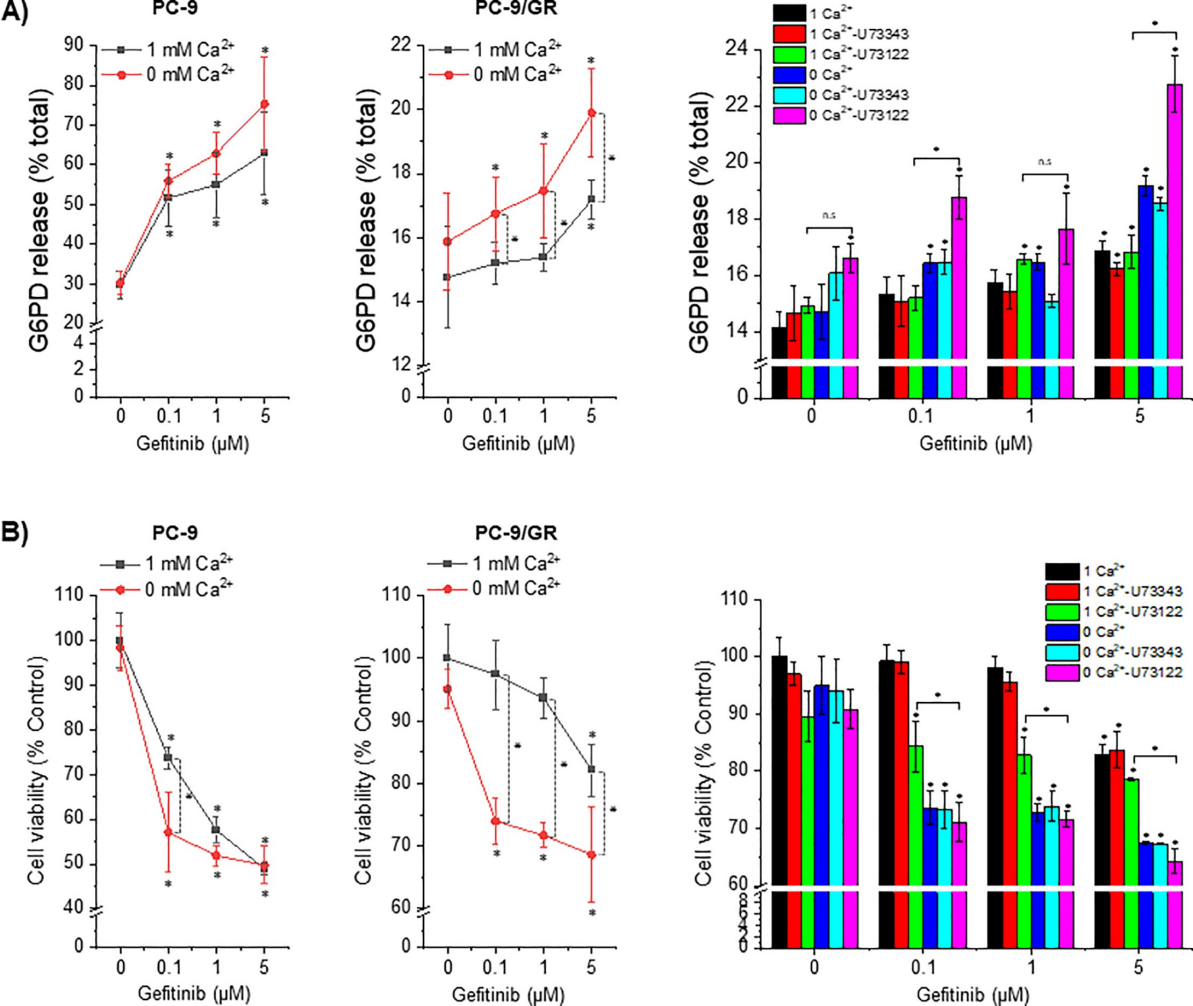
Synergistic effects of low [Ca^2+^]e and U73122 on gefitinib-mediated cytotoxicity. Gefitinib-treated cells with or without [Ca^2+^]e and U73122 were incubated for 24 h and 48 h to evaluate cytotoxicity and cell viability, respectively. (A) Gefitinib-induced cytotoxicity in PC-9 and PC-9/GR cells was evaluated by measuring G6PD activity. Data are presented as proportion of released G6PD (% total; n = 4), and are given as the mean ± S.D. *P < 0.05 compared with control (only vehicle-treated in 1 mM Ca^2+^) group or between indicated groups (B) Cells cultured under indicated conditions were incubated for 48 h and subjected to cell viability assay. Data are presented as relative to control (% control; n = 4) and given as mean ± S.D. *P < 0.05 compared with control (only vehicle-treated in 1 mM Ca^2+^) group or between indicated groups.

Gefitinib reduces EGFR signaling by inhibiting the ATP-binding pocket in the EGFR kinase domain, which sequentially results in cell cycle arrest and apoptosis [17]. As PC-9 and PC-9/GR cells showed identical [Ca^2+^]i oscillations after the removal of [Ca^2+^]e, we examined whether the low [Ca^2+^]e-induced [Ca^2+^]i oscillations were associated with gefitinib-induced cytotoxicity and cell viability. PC-9 and PC-9/GR cells were treated with gefitinib in a dose-dependent manner (0.1, 1, and 5 μM) and incubated in a culture medium supplemented with or without Ca^2+^. After 24- and 48-h incubation periods, cytotoxicity and cell viability, respectively, were evaluated using enzymatic assays. We first showed that gefitinib-treated PC-9 cells exhibited a significant increase in cytotoxicity and decrease in cell viability in a dose-dependent manner regardless of [Ca^2+^]e (left panel, Fig 3A and 3B). However, gefitinib in 1-mM [Ca^2+^]e had no effect on cytotoxicity and cell viability in PC-9/GR cells. Thus, [Ca^2+^]e restriction significantly exacerbated gefitinib-induced cytotoxicity and cell viability compared with the non-gefitinib-treated group and the group treated with gefitinib in 1-mM [Ca^2+^]e (mid panel, Fig 3A and 3B). We investigated the synergistic effects of PLC inhibition and [Ca^2+^]e restriction on cytotoxicity and cell viability. PC-9/GR cells were pretreated with U73122 and U73343 (2 μM), followed by treatment with gefitinib, either in the absence or presence of [Ca^2+^]e. PLC inhibition by U73122 and [Ca^2+^]e restriction synergistically exacerbated cytotoxicity and cell viability (Right panel, Fig 3A and 3B).

### Restricting [Ca^2+^]e in PC-9/GR cells enhances gefitinib-mediated early apoptosis

**Fig 4:**
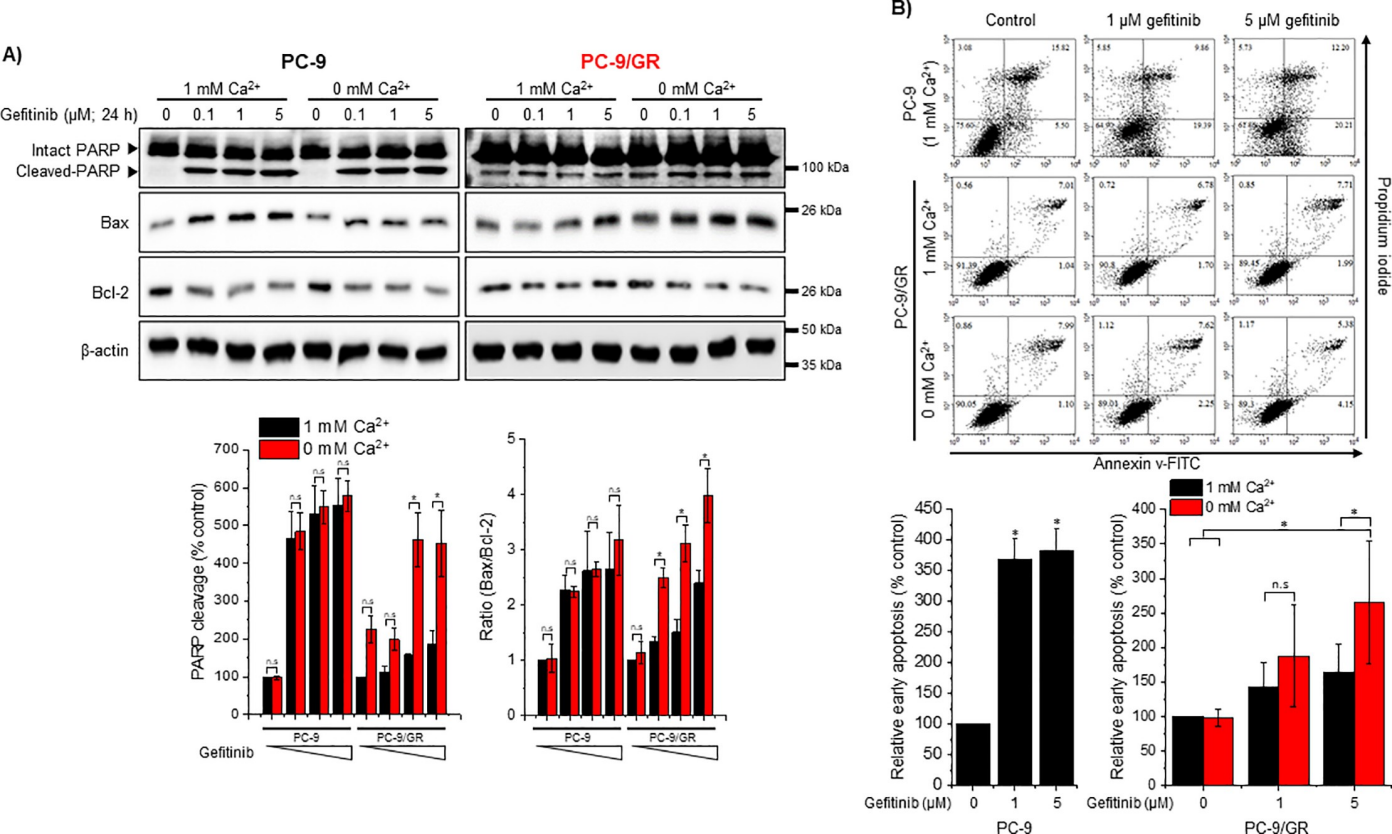
Effects of low [Ca^2+^]e on gefitinib-mediated activation of apoptotic markers. (A) Whole-cell lysates were subjected to western blotting with antibodies to PARP, Bax, Bcl-2, and β-actin. Quantification of PARP cleavage and Bax/Bcl-2 ratio is presented as relative to control (% control; vehicle-treated PC-9 cells in 1 mM [Ca^2+^]e, n ≥ 3). (B) 10,000 cells stained with Annexin V-FITC and PI were evaluated using flow cytometry. Data are percentages compared with control (non-gefitinib-treated) and presented as mean ± S.D. *P < 0.05 compared with control or between indicated groups.

Biochemical assays indicated that gefitinib-mediated cytotoxicity was significantly increased by [Ca^2+^]e restriction in PC-9/GR cells. We investigated the effects of [Ca^2+^]e restriction on the activation of early apoptotic markers. To determine the apoptotic induction, whole-cell lysates were subjected to detect PARP cleavage and Bax/Bcl-2 expression. Gefitinib treatment caused the induction of apoptotic markers in PC-9 cells, irrespective of [Ca^2+^]e (Fig 4A). [Ca^2+^]e restriction in gefitinib-treated PC-9/GR cells resulted in higher PARP cleavage and Bax/Bcl-2 ratio than gefitinib-treated PC-9/GR cells in the presence of [Ca^2+^]e. Next, we performed flow cytometry to quantify early apoptotic cells. [Ca^2+^]e restriction in gefitinib-insensitive cells significantly enhanced early apoptosis in the presence of gefitinib (5 μM) compared to cells treated with the same dose of gefitinib in the presence of [Ca^2+^]e (Fig 4B).

### Restriction of [Ca^2+^]e reduces EGF-mediated NFAT1 induction in gefitinib-resistant cells

**Fig 5:**
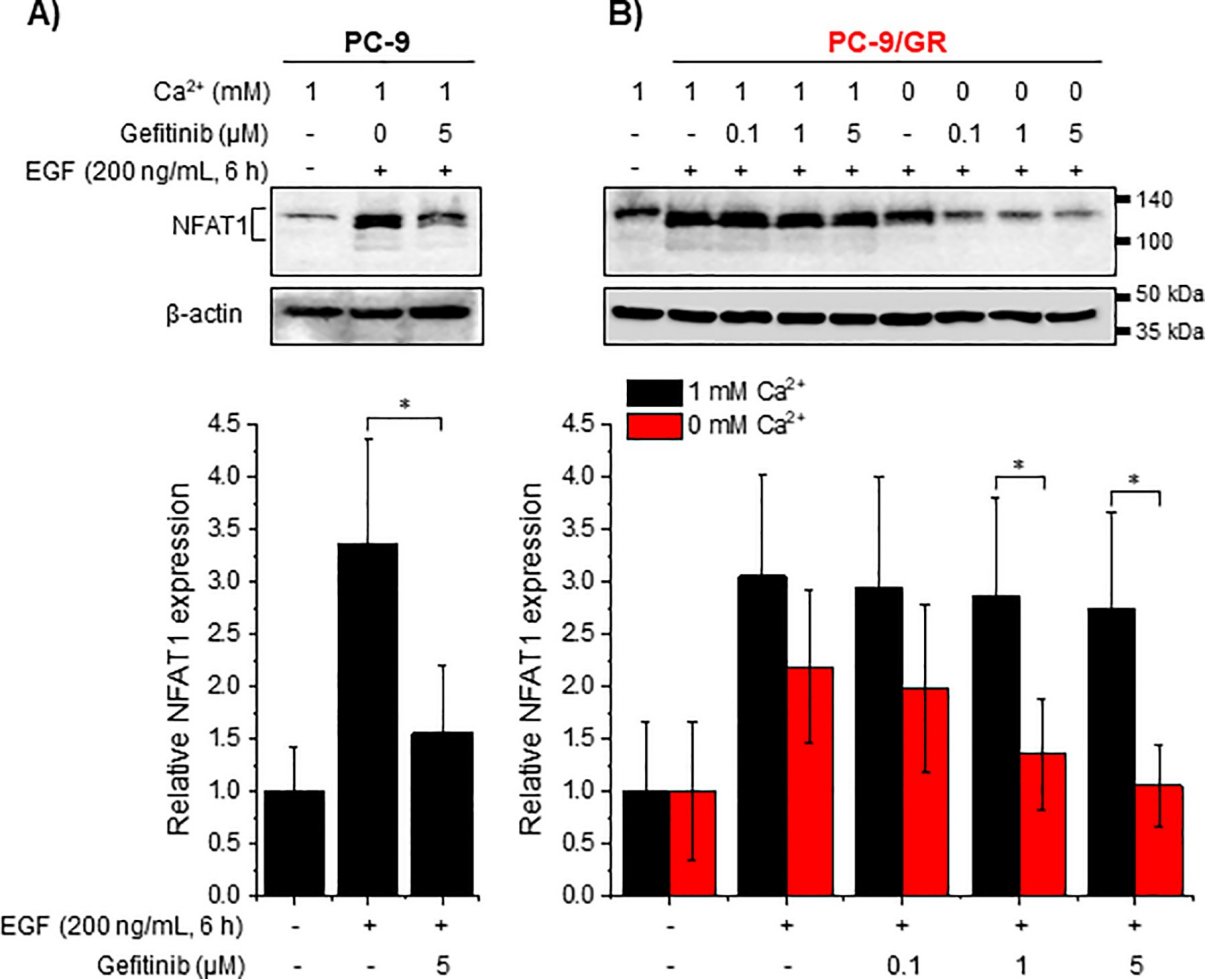
Regulatory effects of low [Ca^2+^]e on EGF-mediated NFAT1 induction. PC-9 and PC-9/GR cells were incubated under the indicated conditions for 6 h, and whole cell lysates were used for determining NFAT1 induction. (A) Inhibition of EGF-mediated NFAT1 induction in PC-9 cells (B) Sustained EGF-mediated NFAT induction and attenuation of EGF-mediated sustained NFAT1 induction in PC-9/GR cells. The column shows the fold-increase of NFAT1 expression compared with control (cells treated without EGF and gefitinib in the presence of [Ca^2+^]e. Data are mean ± S.D. *P < 0.05 compared with control or between indicated groups.

Among the isoforms of NFATs, NFAT1 expression is higher in NSCLC cells and this overexpression is related to the poor survival of patients with NSCLC [18]. This led us to examine whether EGF-stimulated tumor cells elicited NFAT1 induction and whether this was affected by [Ca^2+^]e restriction on gefitinib-resistant cells. Following FBS starvation for 1 h, EGF-treated (200 ng/mL) cells were incubated. EGF-mediated NFAT1 induction was significantly reduced by gefitinib (5 μM) in PC-9 cells (Fig 5A), whereas it was unaffected in gefitinib-treated PC-9/GR cells in the presence of [Ca^2+^]e (Fig 5B). [Ca^2+^]e restriction on gefitinib-treated PC-9/GR cells resulted in the reduction of EGF-mediated NFAT1 induction (Fig 5B). These results indicate that NFAT1 is regulated by EGFR activation and spontaneous [Ca^2+^]e influx is critical for inducing NFAT1 in gefitinib-resistant cells.

## Discussion

Diverse signaling pathways underlying EGFR activation have been implicated in various cellular functions [16]. Increasing evidence shows that altered [Ca^2+^]i signaling plays a key role in tumorigenesis [19]. However, its role in acquired drug resistance is not fully understood. A study has shown that the binding of EGF to EGFR in human glioma cells induces tyrosine kinase-dependent Ca^2+^ oscillations [6]. The expression of sarco/endoplasmic reticulum Ca^2+^-ATPase and IP_3_R in hepatocellular carcinoma cells appears to be lower and higher, respectively, than that in human bronchial epithelial cells, leading to reduced Ca^2+^ content in the ER [20]. Orai3, which mediates SOCE and allows the proliferation and metastasis of abnormal cells, has been reported to be overexpressed in NSCLCs [21]. Moreover, inhibiting Stim1 and Orai1 results in the modulation of tumor migration, metastasis, and proliferation in other cancer cells [22]. Based on these reports, we assumed that EGF-mediated Ca^2+^ signaling is altered depending on acquired drug resistance. Considering that [Ca^2+^]e influx (indicated as F2 in Fig 1B) mediated by ER Ca^2+^ depletion caused no difference in both cell types in our study, increased spontaneous [Ca^2+^]e influx in PC-9/GR cells appeared to be facilitated by low ER Ca^2+^ content. Thus, we showed that gefitinib-resistant cells present EGF-mediated Ca^2+^ signaling differently than gefitinib-sensitive cells. EGF-mediated [Ca^2+^]i oscillations were observed only in gefitinib-sensitive cells. Moreover, basal [Ca^2+^]i levels were more sustained and ER-Ca^2+^ levels were lower in PC-9/GR cells than in PC-9 cells.

PC-9 and PC-9/GR cells showed similar [Ca^2+^]i oscillations following [Ca^2+^]e restriction in the presence of EGF. The low [Ca^2+^]e-mediated [Ca^2+^]i oscillations were abolished by gefitinib even in PC-9/GR cells. In most other cell types, [Ca^2+^]e influx is essential in maintaining agonist-activated [Ca^2+^]i oscillations [23]. Besides maintaining [Ca^2+^]i oscillations in the absence of [Ca^2+^]e, the acute generation of [Ca^2+^]i oscillations following the restriction of [Ca^2+^]e in PC-9/GR cells was unusual. Zanotti et al. reported that low [Ca^2+^]e induces diverse patterns of Ca^2+^ propagation including [Ca^2+^]i oscillations in glial cells [24]. In parathyroid cells, Ca^2+^-sensing receptors detect increased levels of [Ca^2+^]e and induce [Ca^2+^]i signaling, which results in regulating the secretion of parathyroid hormones [25]. Herein, we first report that restricting [Ca^2+^]e on EGF-treated gefitinib-resistant NSCLC cells leads to the induction of [Ca^2+^]i oscillations, which is identical to the [Ca^2+^]i response in gefitinib-sensitive cells. Low [Ca^2+^]e-mediated [Ca^2+^]i oscillations were dependent on the blockade of EGFR activation by gefitinib, even in gefitinib-resistant cells. Considering the lower ER Ca^2+^ content in PC-9/GR cells than in PC-9 cells, cellular organelles, such as lysosomes and mitochondria, might partake in maintaining Ca^2+^ oscillations. However, identifying Ca^2+^ stores that are involved in maintaining [Ca^2+^]i oscillations requires further investigation. We used PC-9/GR cells with secondary mutations [13], which enhanced the affinity of EGFR toward ATP and rendered it gefitinib-resistant. We observed that low [Ca^2+^]e-mediated [Ca^2+^]i oscillations were abolished by gefitinib and U73122 in PC-9/GR and PC-9 cells, suggesting that the absence of [Ca^2+^]e led to enhanced gefitinib sensitivity and reversed gefitinib-mediated cytotoxicity and apoptosis. Mulder et al. demonstrated that initial targeted therapy on PC-9 cells increases the activity of Ca^2+^ signaling-related proteins and deprives the cell of extracellular Ca^2+^, which results in the marked enhancement of afatinib efficacy [12]. Our data indicated that increased [Ca^2+^]e influx in PC-9/GR cells contributed to gefitinib resistance; therefore, restricting [Ca^2+^]e could be key in enhancing drug sensitivity.

The induction of NFATs 1–4 is regulated by [Ca^2+^]i increase, which is mediated by the receptor-activated PLC pathway or extracellular Ca^2+^ influx in immune cells [26]. Inactive NFATs exist in hyper-phosphorylated states in the cytoplasm. Following a receptor-mediated [Ca^2+^]i increase, NFATs are dephosphorylated and activated by Ca^2+^-dependent phosphatases, such as calcineurin [27]. Although still controversial, several studies have demonstrated that NFAT1 exhibits anti-apoptotic properties and promotes tumor progression [28, 29]. Our study indicated that gefitinib-mediated apoptosis and EGF-mediated NFAT1 induction are significantly decreased depending on [Ca^2+^]e restriction in gefitinib-resistant cells. Thus, sustained NFAT1 induction in gefitinib-resistant cells might play a role in acquired drug resistance.

Our study demonstrated that NSCLC cells altered EGF-mediated Ca^2+^ signaling depending on gefitinib resistance. Importantly, restricting [Ca^2+^]e in gefitinib-sensitive and gefitinib-resistant cells elicited identical [Ca^2+^]i oscillations and significantly enhanced gefitinib sensitivity in gefitinib-resistant cells. Moreover, we showed the regulatory effects of [Ca^2+^]e on NFAT1 induction and concluded that restricting [Ca^2+^]e should be used to treat drug-resistant NSCLCs.

## Supporting information

S1 Fig Expression of Orai1, STIM1, and SERCA2 in PC-9 and PC-9/GR cells. PC-9 and PC-9/GR cells were respectively seeded on 60 mm culture dish and cultured in normal condition for overnight. Cells were lysed with RIPA buffer, and collected whole cell lysates were used for Western blot to determine the endogenous expression level of Orai1, STIM1, and SERCA2. Columns present the mean ± S.D. from 3 independent experiments. *P < 0.05.
(TIF)

S1 Raw images (PPTX)
