# Supplementary figures and images for: Restricting extracellular Ca2+ on gefitinib-resistant non-small cell lung cancer cells reverses altered epidermal growth factor-mediated Ca2+ response, which consequently enhances gefitinib sensitivity
Source: PLoS One. 2020 Aug 25;15(8):e0238155. doi: 10.1371/journal.pone.0238155 (PMC7447054; doi:10.1371/journal.pone.0238155)

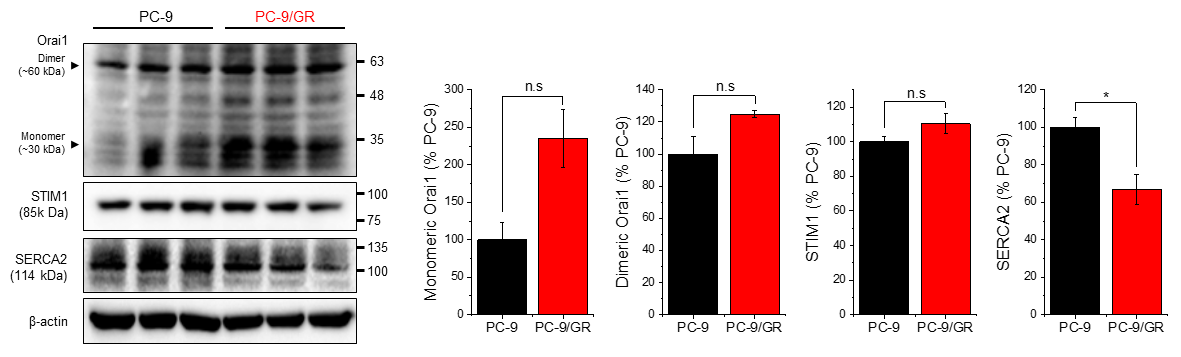

Supplement: S1 Fig — PC-9 and PC-9/GR cells were respectively seeded on 60 mm culture dish and cultured in normal condition for overnight. Cells were lysed with RIPA buffer, and collected whole cell lysates were used for Western blot to determine the endogenous expression level of Orai1, STIM1, and SERCA2. Columns present the mean ± S.D. from 3 independent experiments. *P < 0.05. (TIF) [file pone.0238155.s001.tif]
