# Supplementary material for: Restricting extracellular Ca2+ on gefitinib-resistant non-small cell lung cancer cells reverses altered epidermal growth factor-mediated Ca2+ response, which consequently enhances gefitinib sensitivity
Source: PLoS One. 2020 Aug 25;15(8):e0238155. doi: 10.1371/journal.pone.0238155 (PMC7447054; doi:10.1371/journal.pone.0238155)
Supplement: S1 Raw images — (PPTX) [file pone.0238155.s002.pptx]

## Slide 1
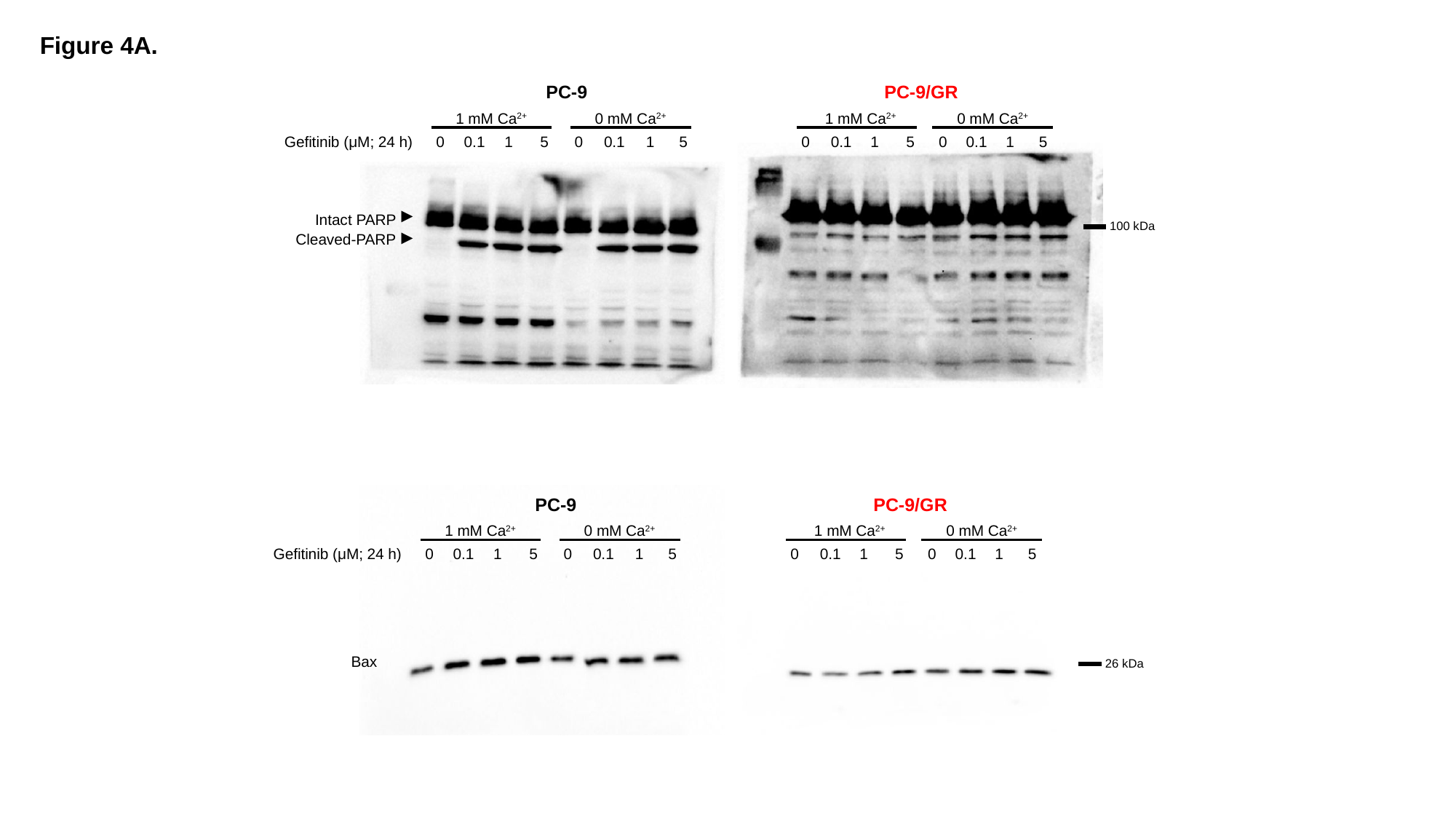

Figure 4A.
PC-9
PC-9/GR
1 mM Ca2+
0 mM Ca2+
1 mM Ca2+
0 mM Ca2+
Gefitinib (μM; 24 h)
0
0.1
1
5
0
0.1
1
5
0
0.1
1
5
0
0.1
1
5
◀
Intact PARP
100 kDa
Cleaved-PARP
◀
PC-9
PC-9/GR
1 mM Ca2+
0 mM Ca2+
1 mM Ca2+
0 mM Ca2+
Gefitinib (μM; 24 h)
0
0.1
1
5
0
0.1
1
5
0
0.1
1
5
0
0.1
1
5
Bax
26 kDa

## Slide 2
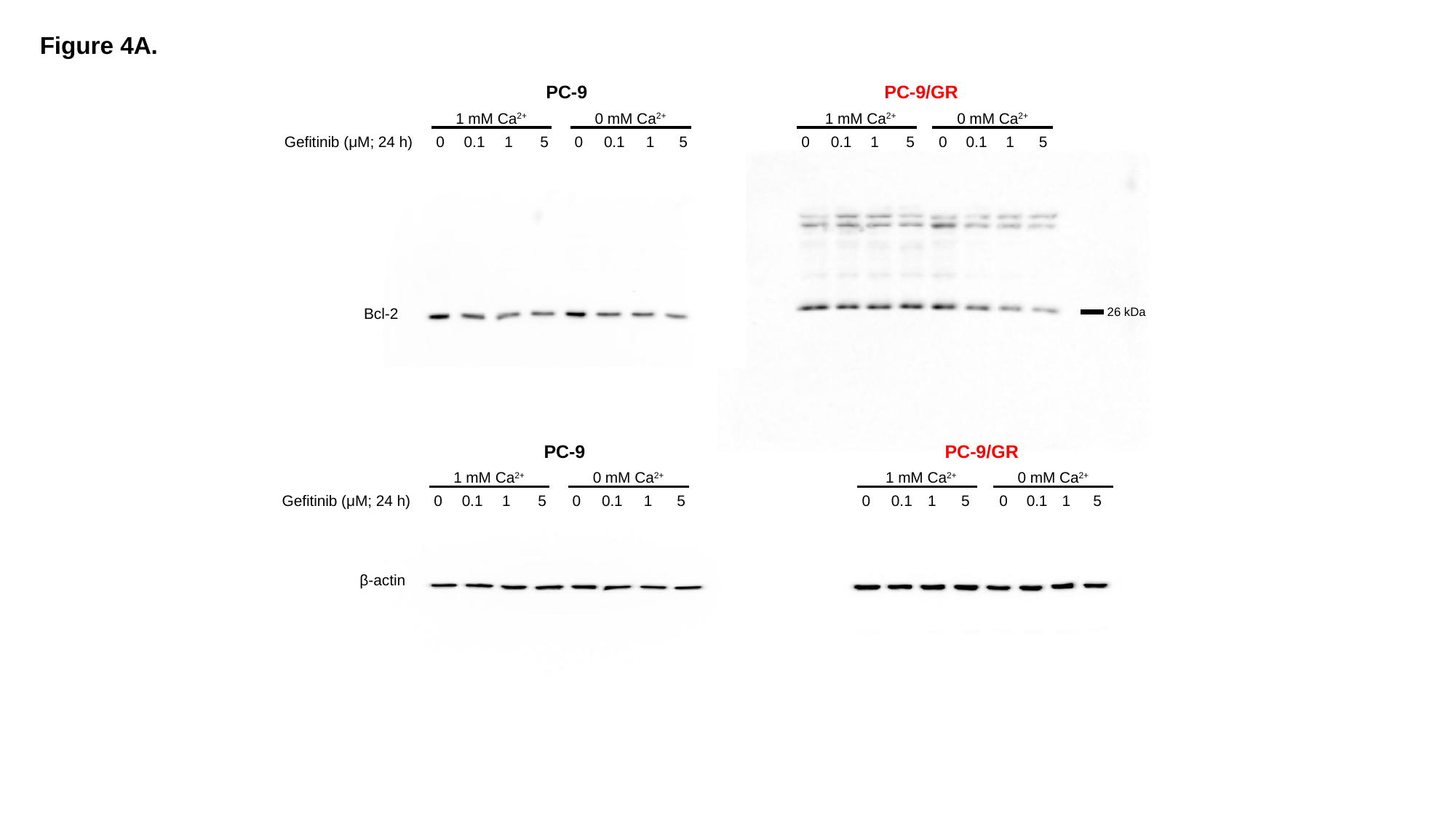

Figure 4A.
PC-9
PC-9/GR
1 mM Ca2+
0 mM Ca2+
1 mM Ca2+
0 mM Ca2+
Gefitinib (μM; 24 h)
0
0.1
1
5
0
0.1
1
5
0
0.1
1
5
0
0.1
1
5
Bcl-2
26 kDa
PC-9
PC-9/GR
1 mM Ca2+
0 mM Ca2+
1 mM Ca2+
0 mM Ca2+
Gefitinib (μM; 24 h)
0
0.1
1
5
0
0.1
1
5
0
0.1
1
5
0
0.1
1
5
β-actin

## Slide 3
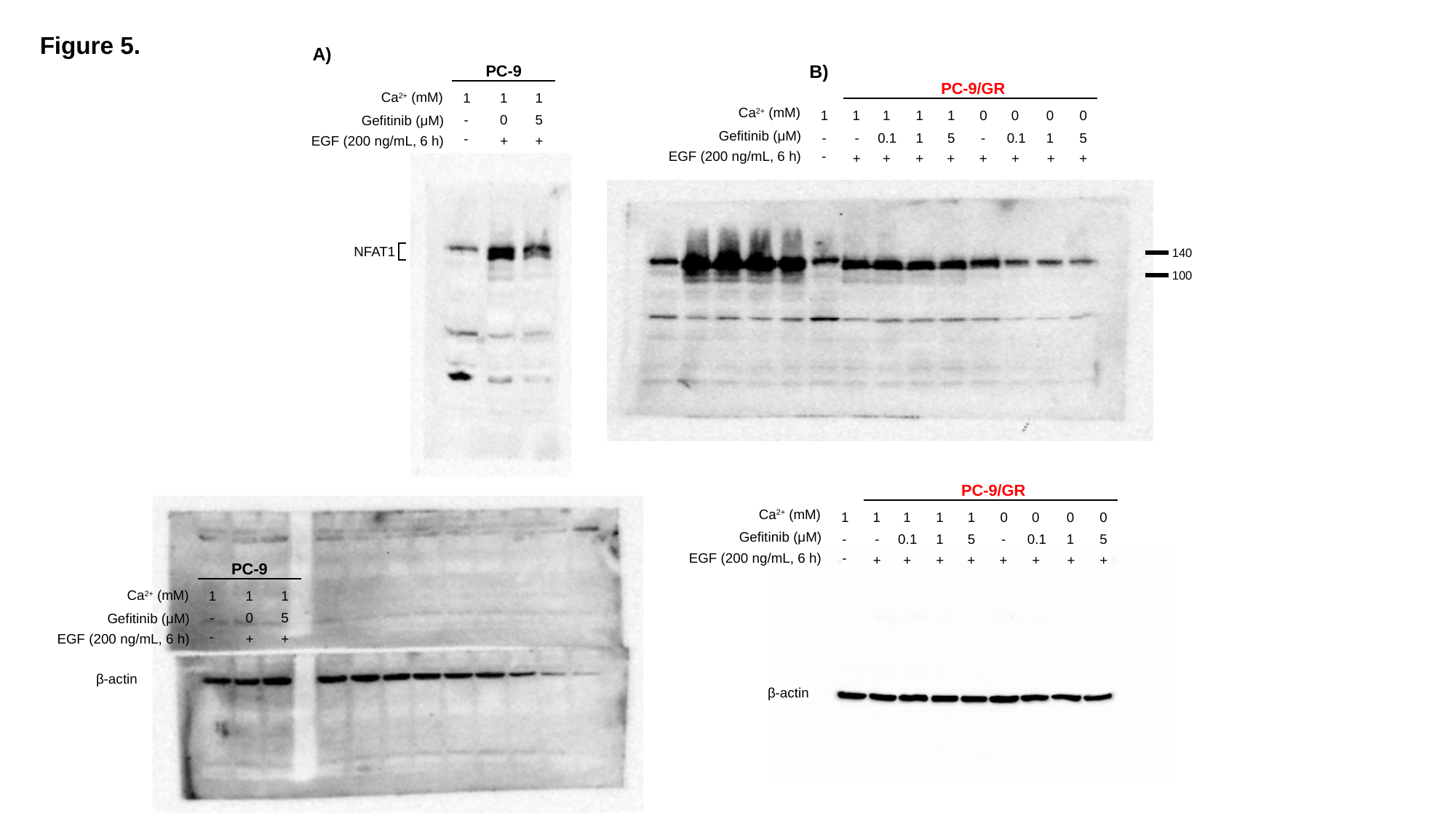

Figure 5.
A)
B)
PC-9
PC-9/GR
Ca2+ (mM)
1
1
1
Ca2+ (mM)
1
1
1
1
1
0
0
0
0
-
0
5
Gefitinib (μM)
Gefitinib (μM)
-
-
0.1
1
5
-
0.1
1
5
-
+
+
EGF (200 ng/mL, 6 h)
EGF (200 ng/mL, 6 h)
-
+
+
+
+
+
+
+
+
NFAT1
140
100
PC-9/GR
Ca2+ (mM)
1
1
1
1
1
0
0
0
0
Gefitinib (μM)
-
-
0.1
1
5
-
0.1
1
5
EGF (200 ng/mL, 6 h)
-
+
+
+
+
+
+
+
+
PC-9
Ca2+ (mM)
1
1
1
-
0
5
Gefitinib (μM)
-
+
+
EGF (200 ng/mL, 6 h)
β-actin
β-actin

## Slide 4
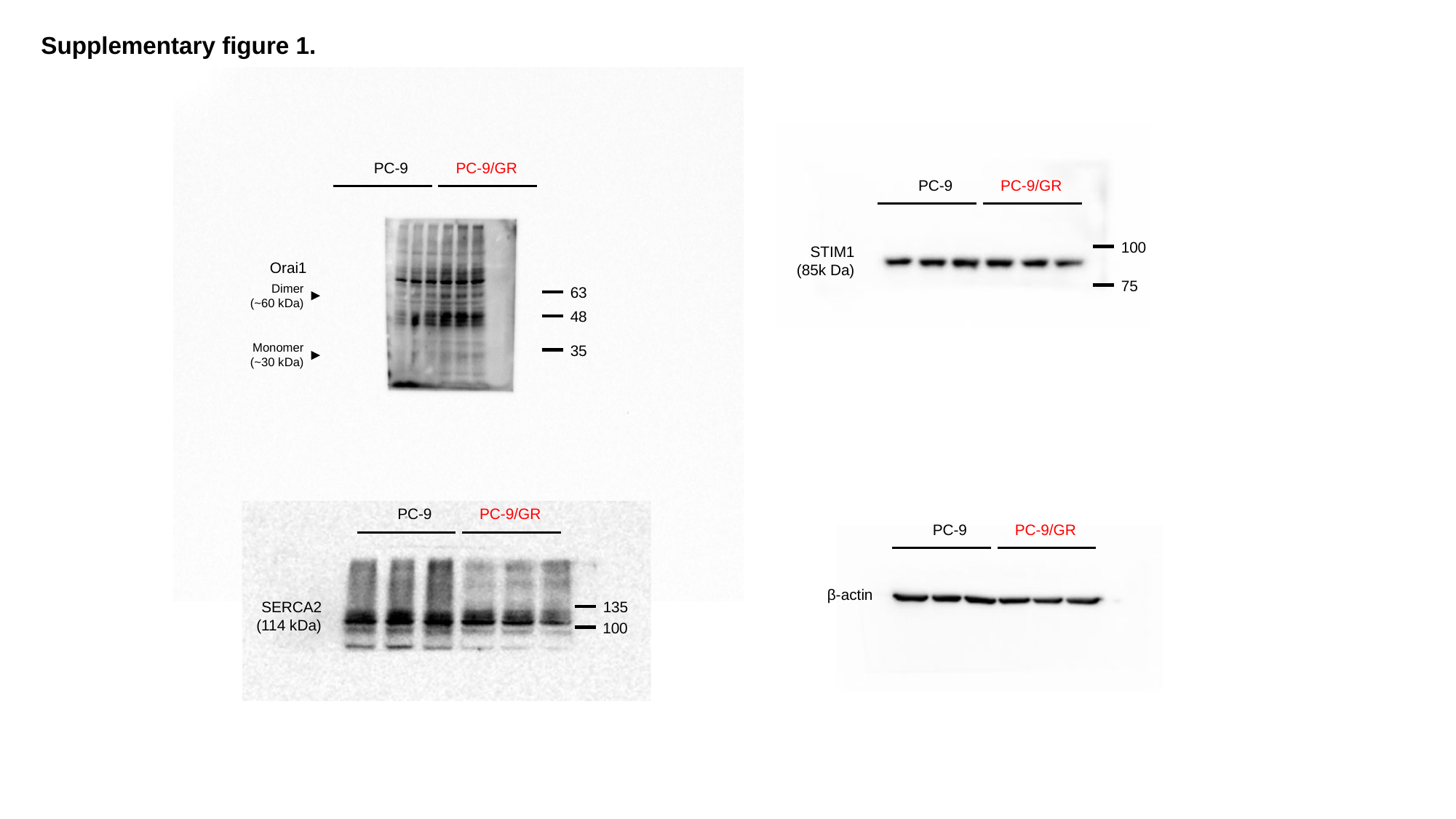

Supplementary figure 1.
PC-9
PC-9/GR
PC-9
PC-9/GR
100
 STIM1
(85k Da)
 Orai1
Dimer
(~60 kDa)
75
63
48
Monomer
(~30 kDa)
35
PC-9
PC-9/GR
PC-9
PC-9/GR
β-actin
135
SERCA2
(114 kDa)
100
